# Modeling how substitution of sedentary behavior with standing or physical activity is associated with health-related quality of life in colorectal cancer survivors

**DOI:** 10.1007/s10552-016-0725-6

**Published:** 2016-02-18

**Authors:** Eline H. van Roekel, Martijn J. L. Bours, José J. L. Breedveld-Peters, Paul J. B. Willems, Kenneth Meijer, IJmert Kant, Piet A. van den Brandt, Geerard L. Beets, Silvia Sanduleanu, Matty P. Weijenberg

**Affiliations:** Department of Epidemiology, GROW School for Oncology and Developmental Biology, Maastricht University, P.O. Box 616, 6200 MD Maastricht, The Netherlands; Department of Human Movement Science, NUTRIM School for Nutrition, Toxicology and Metabolism, Maastricht University, Maastricht, The Netherlands; Department of Epidemiology, CAPHRI School for Public Health and Primary Care, Maastricht University, Maastricht, The Netherlands; Department of Surgery, GROW School for Oncology and Developmental Biology, Maastricht University Medical Center+, Maastricht, The Netherlands; Department of Internal Medicine, Division of Gastroenterology and Hepatology, GROW School for Oncology and Developmental Biology, Maastricht University Medical Center+, Maastricht, The Netherlands

**Keywords:** Isotemporal substitution modeling, Sedentary behavior, Standing, Physical activity, Health-related quality of life, Colorectal cancer survivor

## Abstract

**Purpose:**

Previous research indicates that sedentary behavior is unfavorably associated with health-related quality of life (HRQoL) of colorectal cancer (CRC) survivors. Using isotemporal substitution modeling, we studied how substituting sedentary behavior with standing or physical activity was associated with HRQoL in CRC survivors, 2–10 years post-diagnosis.

**Methods:**

A cross-sectional study was conducted in stage I–III CRC survivors (*n* = 145) diagnosed at Maastricht University Medical Center+, the Netherlands (2002–2010). Sedentary, standing, and physical activity time were measured by the thigh-mounted MOX activity monitor. HRQoL outcomes comprised global quality of life, physical, role, and social functioning, and disability (scales: 0–100), fatigue (20–140), and depression and anxiety (0–21). Isotemporal substitution modeling was applied to analyze associations with HRQoL of substituting sedentary time with equal time in standing or physical activity.

**Results:**

On average, participants spent 10.2 h/day sedentary (SD, 1.7), 3.4 h/day standing (1.3), and 1.7 h/day in physical activity (0.8). In confounder-adjusted isotemporal models, substituting sedentary time with standing or with physical activity was associated with significantly better physical functioning (regression coefficient [β], i.e., difference in outcome score per 1 h/day of sedentary time substituted with standing or physical activity, 3.1; 95 % confidence interval [CI] 0.5, 5.7; and 5.6; 0.7, 10.6, respectively). Substituting sedentary time with standing was also associated with significantly lower disability (β, −3.0; 95 % CI −4.9, −1.1) and fatigue (−4.0; −7.6, −0.3).

**Conclusions:**

Our results suggest that substituting sedentary behavior with standing or physical activity may be beneficially associated with certain HRQoL outcomes in CRC survivors. Prospective studies are warranted to confirm whether actual substitution of sedentary behavior with these activities may improve HRQoL in CRC survivors.

**Electronic supplementary material:**

The online version of this article (doi:10.1007/s10552-016-0725-6) contains supplementary material, which is available to authorized users.

## Introduction

Colorectal cancer (CRC) survivors often experience long-term side effects of the cancer and/or its treatment, such as depressive symptoms and fatigue, which can last for more than ten years after treatment [1]. These persisting problems can negatively influence specific domains of health-related quality of life (HRQoL), such as physical and social functioning [2, 3]. Lifestyle interventions targeting an increase in physical activity and reductions in sedentary behavior (i.e., sitting or lying while awake with a low energy expenditure [4]) may be an avenue to improve the HRQoL of cancer survivors, but little is known on what type of activities could lead to the most optimal health benefits in CRC survivors [5].

Previous prospective research has shown that greater self-reported television viewing time (a specific sedentary behavior) in CRC survivors was negatively associated with HRQoL, in the first three years post-diagnosis [6]. In contrast, a subsequent study in colon cancer survivors that applied objective waist-worn activity monitors to measure sedentary time did not observe any associations with multiple HRQoL outcomes [7]. This inconsistency may be due to differences in measures applied to assess sedentary time within these two studies, including the measurement of one specific sedentary behavior (television watching) by self-report versus more objective measurement by activity monitors of total sedentary time. In addition, the use of a waist-worn monitor in the latter study may have resulted in some degree of measurement error, as these devices infer sedentary behavior from low movement intensity alone, rather than taking also body posture into account. Therefore, this could have resulted in misclassification of stationary standing as sedentary behavior in this study. Thigh-mounted monitors are more suitable to measure sedentary behavior, as these devices are better able to measure posture and can therefore accurately distinguish standing from sitting/lying [8]. This explanation is plausible as we have recently observed that more sedentary time, objectively assessed by thigh-mounted activity monitors, was significantly associated with poorer HRQoL in CRC survivors, 2–10 years post-diagnosis [9]. These findings suggest that reducing sedentary time, which comprises approximately two-thirds of total waking hours within this population [7], might be a promising target for lifestyle interventions aiming to improve the HRQoL of CRC survivors. However, advising individuals to reduce their sedentary time automatically means that this time needs be replaced (substituted) with another type of activity. For development of effective interventions, it is thus important to know which type of activities should replace sedentary behavior, as replacement with different activities can have different associations with health [10].

A growing body of both prospective and cross-sectional evidence shows that moderate-to-vigorous physical activity (MVPA; e.g., brisk walking or swimming) is beneficially related to the HRQoL of CRC survivors [7, 11–18]. However, a high prevalence of comorbidities and old age might make it difficult to perform activities at this intensity for a large proportion of CRC survivors [19]. Interestingly, independent of MVPA, more self-reported time spent in light physical activity (LPA; e.g., light walking or light household work) has also been found to be positively associated with HRQoL outcomes in CRC survivors, such as better physical and role functioning [18, 20]. This indicates that replacing sedentary time with low energy activities, such as standing or light-intensity walking, might be beneficial for CRC survivors’ HRQoL. Therefore, these activities could be a more feasible target for lifestyle interventions to be developed for this population.

Recently, isotemporal substitution modeling has been proposed as an analytic method for analyzing effects of substituting time in one activity for another, while keeping total time and time in other activities constant [10]. These models can be used to assess the effects of replacing time in one behavior (e.g., sitting) with time in other possible behaviors (e.g., standing or physical activity) separately [10]. Using isotemporal substitution modeling, we studied how substituting sedentary behavior with standing or physical activity was associated with HRQoL in CRC survivors, 2–10 years post-diagnosis.

## Materials and methods

### Study design and participants

**Fig. 1:**
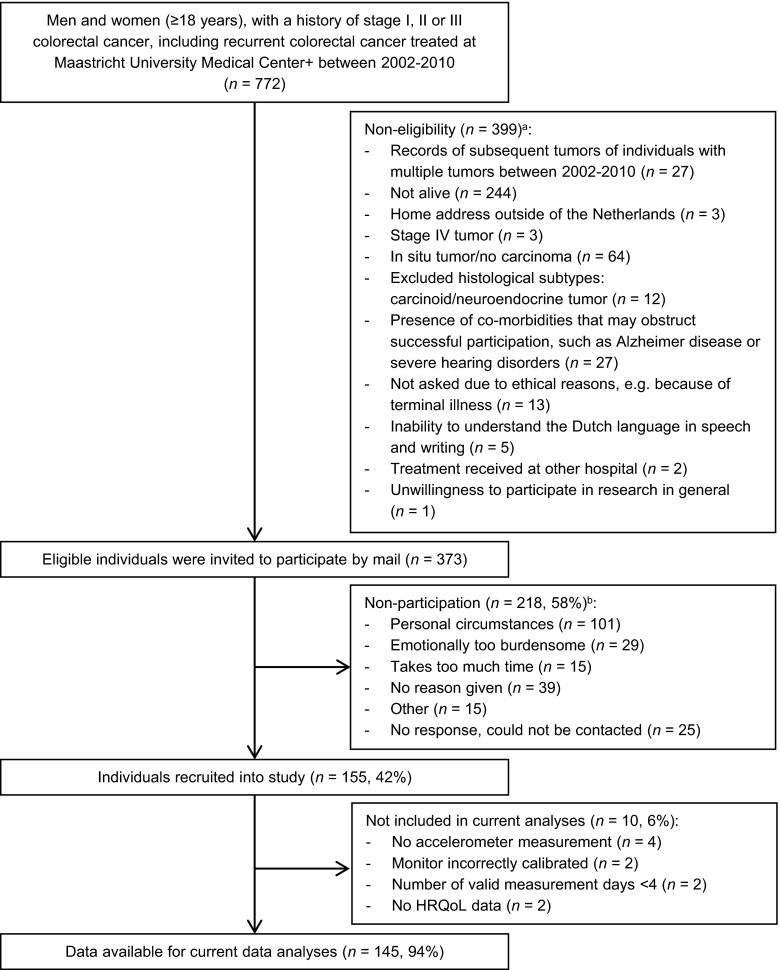
Flow diagram of inclusion of individuals included into the cross-sectional component of the EnCoRe study and analyses presented in this paper. *Footnotes:* ^a^Reasons for non-eligibility are given in order of exclusion, and totals do not add up because some exclusion criteria applied concurrently. ^b^Totals do not add up because some individuals reported multiple reasons for non-participation

Data from the cross-sectional component of the Energy for life after ColoRectal cancer (EnCoRe) study was used. Methods of the EnCoRe study have been described previously [21]. The cross-sectional component was conducted in CRC survivors recruited 2–10 years post-diagnosis. Eligible individuals, i.e., persons diagnosed with and treated for stage I–III CRC between 2002 and 2010 at Maastricht University Medical Center+, the Netherlands, were preselected via the Netherlands Cancer Registry (managed by Comprehensive Cancer Centre the Netherlands). Participants were recruited between May 2012 and December 2013. Reasons for exclusion are shown in Fig. 1. The EnCoRe study had been approved by the Medical Ethics Committee of the Academic Hospital Maastricht and Maastricht University, the Netherlands. Written informed consent was obtained from all participants.

### Data collection

When designing the EnCoRe study, a conceptual model was developed for studying lifestyle and HRQoL in CRC survivors [21], based on the International Classification of Functioning, Disability and Health (ICF) of the World Health Organization [22]. The ICF adopts a broad bio-psychosocial definition of human functioning as a multidimensional concept, which does not only incorporate physical health components (body perspective), but also an individual’s ability to perform his/her daily activities and societal roles (individual and societal perspectives) [23]. Further, it enables identification of environmental and personal factors and the presence of health conditions that can influence functioning. The developed conceptual model [21] was adapted for the current research question to identify relevant variables to be measured and included in our data analyses (Supplementary Fig. 1, Online Resource 1).

#### Sedentary and physical activity time

The triaxial MOX activity monitor (MMOXX1, upgraded version of the CAM monitor) was used for objective measurement of time spent in sedentary behavior, standing, and physical activity (Maastricht Instruments B.V., the Netherlands) [8, 24]. The MOX has a high reproducibility and an excellent validity for estimating time spent in activities and postures in both a controlled laboratory setting (100 % accuracy and Cohen’s kappa of 0.99, compared with direct observation) and in free-living conditions (intraclass correlation coefficient of 0.98, compared with diary records) [8]. The monitor was waterproofed in a finger cot (VWR International B.V., the Netherlands) and attached via hypoallergenic plaster (BSN Medical, the Netherlands) to the anterior thigh 10 cm above the knee. Participants were instructed to wear the monitor 24 h/day on seven consecutive days and to record sleep and any non-wear periods.

A customized MATLAB program (version R2012a, The MathWorks, Inc., USA) was used to classify each 1-second epoch of the data as sedentary (i.e., sitting/lying during waking hours with a low energy expenditure of ≤1.5 metabolic equivalents [METs] [4]), standing (i.e., standing during waking hours with an energy expenditure ≤1.5 METs), or physical activity (i.e., all activities with an energy expenditure >1.5 METs). This classification was done using previously validated thresholds for parameters of motion intensity and orientation of the device [24]. Time in physical activity was not further subdivided according to intensity level into LPA and MVPA, because of limited reproducibility of the monitor for estimating time in activities at a moderate-to-vigorous intensity [8]. Self-reported non-wear and sleeping periods were checked by visualization of triaxial acceleration data, with non-wear time periods adjusted if necessary, and sleeping times determined if missing. Further processing of worn waking data was performed in SAS (version 9.3, SAS Institute Inc., USA). Monitor wear days with ≥10 h of waking wear time were considered valid; only participants with ≥4 valid days were included in the analyses [25]. Sedentary, standing, and physical activity time (h/day) were calculated and averaged across valid measurement days.

#### HRQoL outcomes

Cancer-specific HRQoL was measured using the valid and reliable European Organization for the Research and Treatment of Cancer Quality of Life Questionnaire-Core 30 (EORTC QLQ-C30, version 3.0) [26, 27]. For the subscales global quality of life and physical, role, and social functioning, 100-point scores were calculated [28]. Disability was assessed by the 12-item version of the ICF-based World Health Organization Disability Assessment Schedule II [29], which has good reliability and validity in different populations, including cancer survivors [30, 31]. Fatigue was assessed through the 20-item Checklist Individual Strength, which was originally developed and validated in patients with chronic fatigue syndrome [32, 33], but has also been applied in cancer survivors [34]. The 14-item Hospital Anxiety and Depression Scale was used to determine levels of anxiety and depression [35], which has adequate psychometric properties in cancer patients [36]. By adding scores of individual items within the Checklist Individual Strength and Hospital Anxiety and Depression Scale, scores for fatigue (scale: 20–140), and depression (0–21) and anxiety (0–21) were calculated, with higher scores indicating higher levels of fatigue, depression, and anxiety.

#### Other factors

Socio-demographic characteristics (gender, age, education level, smoking status, paid employment) and the presence of a stoma were self-reported. Body height and weight were measured by trained personnel for calculation of body mass index (BMI, kg/m^2^). The number of comorbidities was assessed using the 13-item Self-Administered Comorbidity Questionnaire [37]. Perceived deficiency in social support (scale: 6–18) was measured by the six-item Dutch Social Support List (SSL-6) [38]. Clinical characteristics (cancer stage, age at diagnosis, treatment, and tumor subsite) were collected through the Netherlands Cancer Registry.

### Statistical analyses

Descriptive statistics were calculated for socio-demographic and clinical factors in survivors included and not included in the analyses and for accelerometer-derived characteristics and HRQoL outcomes in included individuals stratified by gender. Multivariable linear regression models were used to analyze associations of standing and physical activity time (h/day) with HRQoL outcomes. Unstandardized regression coefficients (β) with 95 % confidence intervals (CIs) were calculated, representing differences in mean HRQoL scores per additional 1 h/day of standing or physical activity, which was similar to one standard deviation (SD) of these variables within the sample. Potential confounding factors included in multivariable models were selected a priori from our ICF-based conceptual model (Supplementary Fig. 1, Online Resource 1). These were either adjusted for in all models (age, gender, number of comorbidities, years since diagnosis, cancer stage, smoking status, and BMI) or, only when retained via backward elimination using *p* > 0.2 as a cutoff for exclusion [39] (education level, paid employment, having a partner, the presence of a stoma, radiotherapy and/or chemotherapy treatment, tumor subsite, and perceived deficiency in social support). None of the models showed evidence for multicollinearity (variance inflation factors ≤5 [40]).

Three types of regression models were fitted to analyze associations of standing and physical activity with selected HRQoL outcomes [10]. All models were adjusted for a similar confounder set, but differed with regard to the inclusion of activity variables. First, single-variable models were conducted, which included only one activity variable (sedentary, standing, or physical activity time), thereby estimating *overall* associations of these activity categories with HRQoL outcomes separately. Secondly, partition models were fitted which included all activity variables (i.e., sedentary, standing, and physical activity time) in one model, to assess *independent* associations of each activity category with the outcome, while keeping time in other activity categories constant.

Third, isotemporal substitution models were fitted for estimating associations with HRQoL of replacing (substituting) time in one category (e.g., sedentary time) with equal time in another category (e.g., standing), while keeping total time and time in the remaining category (e.g., physical activity) constant. Detailed explanation of these models has been published previously [10]. To address our main research question of estimating associations of substituting sedentary time with standing or physical activity, standing and physical activity time were included in the isotemporal model and sedentary time was left out, while the model was adjusted for total waking wear time (i.e., total time was held constant). By constraining the total amount of time, an increase of 1 h/day in standing time implies substitution of the left-out variable (i.e., 1 h/day less sedentary time) with standing, while holding physical activity time constant. As a result, βs from the standing and physical activity time variables represent differences in mean HRQoL scores associated with substituting 1 h/day of sedentary time with equal time in standing or physical activity, respectively. These isotemporal substitution models were considered our main analyses. Similarly, as an additional analysis, we also assessed isotemporal associations of substituting standing time with physical activity, by including sedentary and physical activity time in the models and leaving out standing time.

Minimum differences of interest were defined and based on minimally important differences for the HRQoL outcomes, i.e., published “medium” differences for the EORTC QLQ-C30 subscales [41], and 0.5 times the SD of the score for other outcomes [42] (disability, fatigue, depression, and anxiety). We defined the association to be “meaningful” if the difference in HRQoL outcome associated with a difference of two SDs in the substituted activity variable (i.e., sedentary time or standing time) exceeded these minimum important differences. Otherwise, the association was described as “small.” As the regression coefficients represented the difference in HRQoL outcome score per 1 h/day of the substituted variable, we rescaled the minimum important differences into cutoffs that could be directly compared with the regression coefficients reported, based on this definition. This was done by dividing each of the minimum important differences by two SDs of the substituted activity variable. The cutoffs that were calculated accordingly are shown in Supplementary Table 1 (Online Resource 1). Potential effect modification by gender, age (<70 vs ≥70 years), number of comorbidities (≥2 vs <2), BMI (<30 vs ≥30 kg/m^2^), and perceived deficiency in social support (no deficiency [six-item Social Support List score = 6] vs deficiency [score > 6] [38]) was explored by performing subgroup analyses. To avoid over-interpretation of spurious findings, results were reported if a meaningful and significant association in a certain direction was observed in multiple HRQoL outcomes in one subgroup, but not in the other subgroup.

As HRQoL outcomes were not normally distributed, findings were verified in isotemporal logistic regression models with dichotomized outcomes using gender-specific medians as cutoff [43]. All analyses were performed using IBM SPSS Statistics (version 22, IBM Corporation, USA), and *p* < 0.05 (two-tailed) was considered statistically significant.

## Results

### Participant characteristics

A total of 373 eligible CRC survivors were invited to participate, of whom 155 were recruited (response rate, 42 %; Fig. 1), and 145 were included in data analyses. A total of 10 recruited CRC survivors were excluded from current data analyses, because no accelerometer measurement was performed (*n* = 4), the monitor was incorrectly calibrated (*n* = 2), the number of valid measurements days was <4 (*n* = 2), or no HRQoL data were obtained (*n* = 2). Included participants, compared with eligible survivors not included (Table 1), were significantly younger (difference, 3.5 years; *p* = 0.001), but not significantly different in time since diagnosis (difference, 0.01 years), and gender, tumor subsite, treatment, and cancer stage (differences, <10 %).

**Table 1:** Socio-demographic and clinical characteristics of eligible colorectal cancer survivors included and not included in the current analyses

Characteristic	Included in analyses (*n* = 145)		Not included in analyses (*n* = 228)		*p* ^a^
	*n*	%	*n*	%	
Age (years)					<.01
Mean	70.0		73.4		
SD	8.7		11.8		
Years since diagnosis					.97
Mean	5.7		5.7		
SD	1.9		1.6		
Gender					.35
Men	91	62.8	132	57.9	
Women	54	37.2	96	42.1	
Tumor subsite					.46
Colon	78	53.8	137	60.1	
Rectosigmoid	7	4.8	8	3.5	
Rectum	60	41.4	83	36.4	
Cancer stage^b^					.84
I	40	29.2	58	26.4	
II	50	36.5	85	38.6	
III	47	34.3	77	35.0	
Treatment with surgery					.55
Yes	139	95.9	222	97.4	
No	6	4.1	6	2.6	
Treatment with chemotherapy					.14
Yes	75	51.7	100	43.9	
No	70	48.3	128	56.1	
Treatment with radiotherapy					.18
Yes	55	37.9	71	31.1	
No	90	62.1	157	68.9	
Number of comorbid conditions					
None	35	24.1			
1	36	24.8			
≥2	74	51.0			
Stoma (colostomy/ileostomy)					
Yes	24	16.6			
No	121	83.4			
Body mass index (kg/m^2^)					
Mean	27.6				
SD	4.3				
Education level					
Low	37	25.5			
Medium	48	33.1			
High	60	41.4			
Smoking status					
Current	16	11.0			
Former	98	67.6			
Never	31	21.4			
Perceived deficiency in social support^c^					
Yes	64	44.4			
No	80	55.6			
Paid employment					
Yes	24	16.6			
No	121	83.4			

*SD* standard deviation

^a^Testing differences in characteristics between included and not included eligible colorectal cancer survivors if data were available for both groups; by Pearson’s Chi-square test for most categorical variables (gender, tumor subsite, tumor stage, and treatment with radiotherapy and chemotherapy), Fisher’s exact test for treatment with surgery (due to expected frequency below 5 in one cell), and independent *t* test for continuous variables (age and years since diagnosis)

^b^Data missing for 16 cases (eight included and eight excluded survivors)

^c^Data missing for one participant; dichotomized based on six-item Social Support List score (scale: 6–18, with higher score indicating higher deficiency); categorized into no deficiency (score = 6) vs deficiency (score > 6)

Participants (63 % men, Table 1) had a mean age of 70.0 years (SD, 8.7) and were on average 5.7 years since CRC diagnosis (SD, 1.9). Of all included survivors, 54 % had a history of colon cancer and 41 and 5 % of a rectum and rectosigmoid tumor, respectively. Most participants were either overweight (BMI 25–30 kg/m^2^, 46 %) or obese (BMI ≥ 30 kg/m^2^, 28 %). Approximately half (51 %) of all participants reported at least two comorbidities. Accelerometer data showed that participants spent on average 10.2 h/day sedentary (SD, 1.7, Table 2), 3.4 h/day standing (SD, 1.3), and 1.7 h/day in physical activity (SD, 0.8). The average number of valid accelerometer wear days was 6.8 (SD, 0.6), and the mean waking wear time was 15.3 h/day (SD, 0.8). Men had a higher mean sedentary time (10.5 vs 9.6 h/day) and lower mean standing time (3.2 vs 3.8 h/day) than women, but mean physical activity time was similar between genders. Sedentary time was inversely correlated with standing (Pearson’s *r*, −0.8; *p* < 0.001) and physical activity time (r, −0.6; *p* < 0.001), while standing and physical activity time were positively correlated (*r*, 0.4; *p* < 0.001).

**Table 2:** Descriptive statistics for accelerometer data and health-related quality of life outcome scores by gender in included colorectal cancer survivors

	Men (*n* = 91)		Women (*n* = 54)		Total (*n* = 145)	
	Mean	SD	Mean	SD	Mean	SD
Accelerometer data						
Number of valid days	6.8	0.5	6.7	0.6	6.8	0.6
Waking wear time, h/day	15.4	0.7	15.2	1.0	15.3	0.8
Sedentary time, h/day	10.5	1.5	9.6	1.8	10.2	1.7
Standing time, h/day	3.2	1.1	3.8	1.5	3.4	1.3
Physical activity time, h/day	1.7	0.7	1.7	0.8	1.7	0.8
Health-related quality of life outcomes (scale)^a^						
Global quality of life (0–100)	79.4	15.9	74.5	21.6	77.6	18.3
Physical functioning (0–100)	84.2	18.5	74.8	22.7	80.7	20.6
Role functioning (0–100)	87.0	22.2	77.5	29.0	83.4	25.3
Social functioning (0–100)	88.8	20.2	90.1	14.7	89.3	18.3
Disability (0–100)^b^	10.5	13.8	16.0	17.9	12.5	15.6
Fatigue (20–140)^c^	55.0	25.3	58.1	30.4	56.1	27.2
Depression (0–21)^d^	4.4	3.5	3.9	3.2	4.2	3.4
Anxiety (0–21)^d^	3.9	3.4	4.7	3.8	4.2	3.5

*SD* standard deviation

^a^Higher scores indicate higher global quality of life, physical, role, and social functioning, disability, fatigue, depression, and anxiety

^b^Data missing for four participants (one man and three women)

^c^Data missing for two participants (both women)

^d^Data missing for one participant (man)

### Single-variable and partition models

Results of single-variable models showed significant associations of both standing and physical activity with better physical functioning and lower disability and also of standing with better role functioning and lower fatigue scores (Table 3). These associations were similar within partition models, although mostly attenuated and less significant (Table 3).

**Table 3:** Associations of sedentary, standing, and physical activity time with health-related quality of life scores^a^ in colorectal cancer survivors in single-variable and partition linear regression models^b^

	Sedentary (per 1 h/day)		Standing (per 1 h/day)		Physical activity (per 1 h/day)	
	β	95 % CI	β	95 % CI	β	95 % CI
Global quality of life (*n* = 136)						
Single-variable models^c^	−1.6	−3.4, 0.1	1.2	−1.0, 3.5	3.6	−0.8, 7.9
Partition model^d^	−1.7	−5.2, 1.8	−0.8	−4.5, 2.9	1.4	−4.4, 7.3
Physical functioning (*n* = 136)						
Single-variable models^c^	−3.3	−5.2, −1.4	4.4	2.1, 6.8	8.3	3.7, 13.0
Partition model^d^	1.0	−2.6, 4.6	4.1	0.3, 7.9	6.6	0.6, 12.7
Role functioning (*n* = 136)						
Single-variable models^c^	−2.4	−4.8, 0.0	3.5	0.5, 6.5	4.3	−1.8, 10.3
Partition model^d^	0.2	−4.7, 5.1	3.3	−1.8, 8.4	1.9	−6.1, 9.9
Social functioning (*n* = 136)						
Single-variable models^c^	−1.0	−2.9, 0.8	1.1	−1.2, 3.4	1.2	−3.4, 5.8
Partition model^d^	−1.0	−4.7, 2.7	0.2	−3.7, 4.1	−0.6	−6.8, 5.5
Disability (*n* = 132)						
Single-variable models^c^	2.7	1.4, 4.1	−3.7	−5.4, −2.0	−5.8	−9.2, −2.4
Partition model^d^	−0.3	−2.9, 2.4	−3.2	−6.1, −0.4	−3.6	−8.0, 0.8
Fatigue (*n* = 134)						
Single-variable models^c^	3.6	1.0, 6.2	−4.3	−7.5, −1.0	−6.1	−12.6, 0.4
Partition model^d^	1.8	−3.3, 6.8	−2.2	−7.5, 3.2	−1.8	−10.3, 6.6
Depression (*n* = 135)						
Single-variable models^c^	0.2	−0.2, 0.5	−0.1	−0.6, 0.3	−0.5	−1.3, 0.3
Partition model^d^	0.1	−0.6, 0.7	0.0	−0.7, 0.7	−0.4	−1.5, 0.7
Anxiety (*n* = 135)						
Single-variable models^c^	0.1	−0.2, 0.5	−0.1	−0.6, 0.3	−0.7	−1.5, 0.2
Partition model^d^	−0.1	−0.8, 0.6	0.0	−0.8, 0.7	−0.8	−1.9, 0.4

*β* unstandardized regression coefficient (representing the difference in mean health-related quality of life score per additional 1 h/day of sedentary, standing or physical activity time);* CI* confidence interval

^a^Scales are 0–100 (global quality of life, physical, role, and social functioning, and disability), 20–140 (fatigue), and 0–21 (depression and anxiety), with higher scores indicating higher global quality of life, physical, role, and social functioning, disability, fatigue, depression, and anxiety

^b^All models were adjusted for age (years), gender, number of comorbidities (0/1/≥ 2), smoking status (current/previous or never), time since diagnosis (years), cancer stage (I/II/III), body mass index (kg/m^2^), perceived deficiency in social support score (continuous), chemotherapy received (yes/no; only models with physical functioning, fatigue, and depression as outcome), stoma (yes/no; only models with physical and role functioning, disability, and anxiety as outcome), tumor subsite (colon/rectum, with rectosigmoid classified as rectum; only models with physical and role functioning, and disability as outcome), education level (low/medium/high; only models with fatigue and depression as outcome), having a partner (yes/no; only models with anxiety as outcome)

^c^Each activity category (sedentary, standing, and physical activity time) was entered separately in a single confounder-adjusted model without adjustment for any of the other activities, to estimate overall associations of each activity category separately

^d^All activity categories (sedentary, standing, and physical activity time) were entered simultaneously in a single confounder-adjusted model, to estimate independent associations of each activity category, while keeping time in other activities constant

### Isotemporal substitution models

**Fig. 2:**
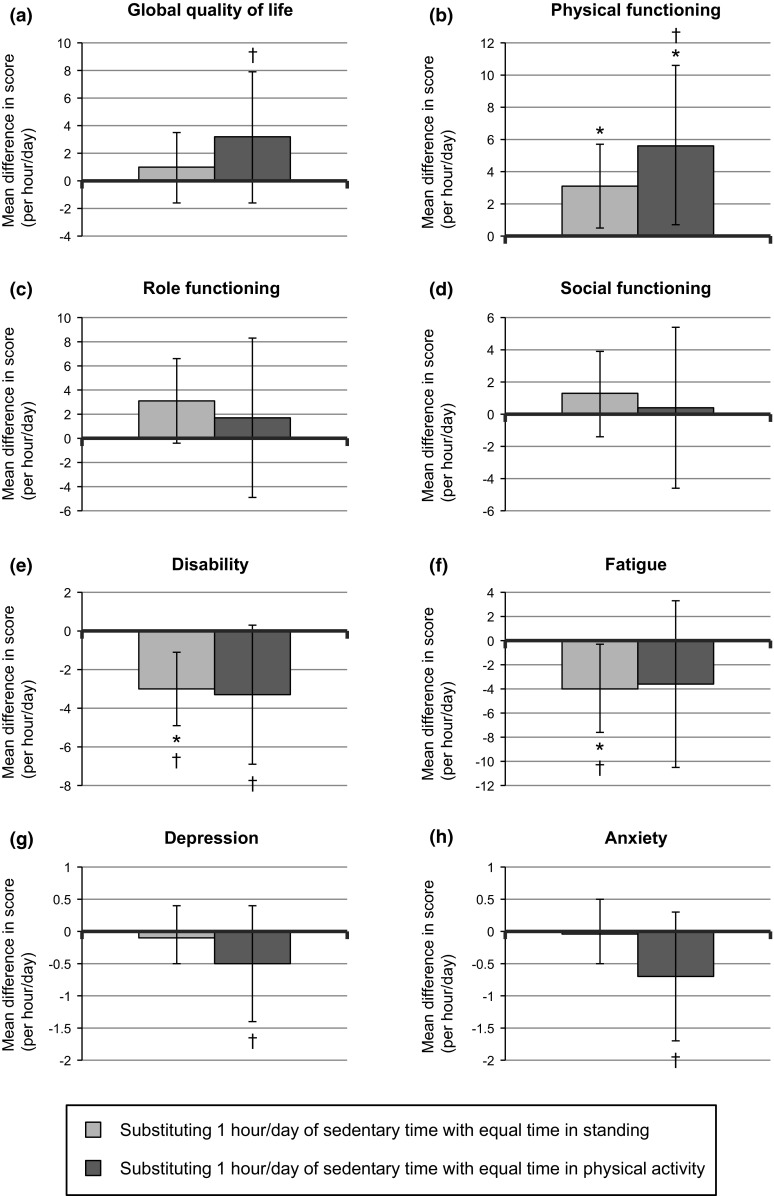
Results of confounder-adjusted isotemporal substitution models for investigating associations of substituting 1 h/day of sedentary time with equal time in standing or physical activity with **a** global quality of life, **b** physical, **c** role, and **d** social functioning (European Organization for the Research and Treatment of Cancer Quality of Life Questionnaire-Core 30, *n* = 136, scales: 0–100), **e** disability (World Health Organization Disability Assessment Schedule II, *n* = 132, scale: 0–100), **f** fatigue (Checklist Individual Strength, *n* = 134, scale: 20–140), and **g** depression and **h** anxiety (Hospital Anxiety and Depression Scale, *n* = 135, scales: 0–21). *Dagger* (†) denotes that the mean difference in health-related quality of life score (i.e., the regression coefficient) exceeds calculated cutoff for meaningful differences in health-related quality of life outcomes, and *asterisk* (*) denotes that the mean difference is statistically significant (*p* < 0.05)

Within isotemporal substitution models modeling associations of substitution of sedentary behavior with standing or physical activity on HRQoL outcomes (Fig. 2; Supplementary Table 2, Online Resource 2 with detailed results), a significantly higher physical functioning score was observed for substituting 1 h/day of sedentary time with standing (β, 3.1; 95 % CI 0.5, 5.7) or with physical activity (β, 5.6; 95 % CI 0.7, 10.6). In addition, substituting 1 h/day of sedentary time with standing was significantly associated with lower disability and fatigue scores (β, −3.0; 95 % CI −4.9, −1.1; and −4.0; −7.6, −0.3, respectively). All statistically significant associations were also of a meaningful magnitude (Supplementary Table 1, Online Resource 2), except for the association between substituting sedentary time with standing and physical functioning. Further, favorable associations that exceeded cutoffs for medium effect sizes were found for substituting 1 h/day of sedentary time with equal time in physical activity with global quality of life, disability, depression, and anxiety, but these associations were not statistically significant (Fig. 2; Supplementary Table 2, Online Resource 2). Nonsignificant and generally small associations were observed between substituting standing time with physical activity and HRQoL outcomes (Supplementary Table 2, Online Resource 2). Isotemporal logistic regression models with dichotomized outcomes showed generally comparable results, but with wider CIs (Supplementary Table 3, Online Resource 2).

### Subgroup analyses

We observed differences in results between subgroups based on gender, age, BMI, and perceived deficiency in social support (Supplementary Table 4, Online Resource 2). In women only, substituting sedentary time with physical activity was associated with significantly better physical functioning and lower disability scores. Further, only in survivors below 70 years of age, substituting sedentary time with standing was associated with significantly higher physical functioning and lower disability scores. In addition, only in non-obese survivors, substituting sedentary time with physical activity was associated with significantly higher global quality of life, and lower depression and anxiety scores, and substituting standing time with physical activity was associated with lower depression and anxiety scores. Finally, only in survivors reporting a deficiency in social support, substituting sedentary time with standing was associated with significantly better global quality of life and physical functioning, and lower disability and fatigue scores. No differences in results by number of comorbidities were found.

## Discussion

To our knowledge, this is the first study applying isotemporal substitution modeling to study how substituting sedentary time with standing or physical activity was associated with HRQoL outcomes in CRC survivors. Our results show that substituting 1 h/day of sedentary time with equal time in standing or physical activity was associated with significantly better physical functioning. In addition, substitution of sedentary time with standing was associated with significantly lower disability and fatigue. Nearly all of these significant associations were of a meaningful magnitude, which suggests that these findings may have clinical relevance. Substituting sedentary time with physical activity was found to be meaningfully but not significantly associated with better global quality of life, and lower disability, depression, and anxiety. We observed neither significant nor meaningful associations with role and social functioning.

In line with our findings, a previous prospective study, which applied isotemporal substitution modeling for analyzing effects of substituting sedentary time with LPA and MVPA in older adults from the general population, observed that substitution with LPA or with MVPA was independently associated with better self-reported physical health, while LPA was also associated with psychosocial well-being [44]. One hypothesized mechanism linking sedentary behavior with health-related outcomes in cancer survivors is metabolic dysfunction, in particular adiposity and insulin resistance [45]. Previous prospective studies applying isotemporal modeling have observed that substituting sedentary behavior with LPA and MVPA was beneficially associated with cardio-metabolic outcomes in the general population [46] and weight loss in premenopausal women [10]. In addition, an intervention study in a controlled laboratory setting with 18 healthy subjects recently observed that substituting 6 h/day of sedentary time with 4 h of walking and 2 h of standing significantly improved insulin sensitivity and plasma lipids [47]. This further suggests that replacing sedentary time with time in LPA, such as standing or walking, might be beneficial for metabolic health, next to increasing levels of MVPA. Whether these mechanisms can explain the associations we observed with HRQoL in CRC survivors remains to be elucidated.

An important strength of our study is the use of isotemporal substitution modeling, which enabled us to analyze separately how substituting sedentary time with standing or with physical activity was associated with the HRQoL of CRC survivors. In addition, the use of a thigh-mounted activity monitor provided objective and accurate data on sedentary, standing, and physical activity time [8, 24]. However, due to the limited reproducibility of the MOX monitor at moderate-to-vigorous intensity levels, we could not differentiate between LPA and MVPA within our analyses. Another limitation to consider is the cross-sectional design of our study. We cannot exclude the possibility of reverse causality, and our substitution models represent cross-sectional replacements of time spent in different activities on a population level, rather than actual activity replacement within individuals. Even though the association of sedentary behavior with HRQoL in CRC survivors is likely to be reciprocal and to result in a downward spiral, interventions targeting sedentary behavior might break this spiral and thus improve HRQoL in CRC survivors [5]. Prospective data are needed to confirm our findings. Additionally, participants differed in age and perhaps other (non-measured) characteristics from non-participants, which could limit the generalizability of our findings due to potential selection bias. Further, we performed a considerable number of significance tests within our analyses, which may have resulted in false positive findings. However, we observed a consistent pattern of substitution of sedentary behavior with standing or physical activity being beneficially associated with multiple HRQoL outcomes according to a priori hypothesized directions and mostly exceeding predefined cutoffs for meaningful differences. This suggests that the observed associations are not likely to be mere chance findings but could be clinically relevant associations, although replication in future studies is necessary. Finally, due to the limited sample size, our statistical analyses might have been underpowered for detecting associations of substituting sedentary time with physical activity, as meaningful but nonsignificant associations were observed with certain HRQoL outcomes.

In conclusion, we observed in these cross-sectional analyses using isotemporal substitution modeling that substituting sedentary time with standing or with physical activity may be beneficially associated with certain HRQoL outcomes in CRC survivors. We observed that certain associations differed depending on specific characteristics of CRC survivors, such as age and gender, which could be relevant for development of tailored lifestyle interventions aimed at safeguarding this population’s HRQoL. Prospective studies are needed to examine whether actual replacement of sedentary time with standing or with physical activity can lead to clinically relevant improvements in the HRQoL of CRC survivors and to reveal the underlying mechanisms. This information can be used to develop more effective lifestyle interventions targeting activities which likely have the most optimal health benefits for CRC survivors. Ultimately, these interventions will be suitable candidates for further testing in future intervention studies.

## Electronic supplementary material

Supplementary material 1 (PDF 147 kb)

Supplementary material 2 (PDF 142 kb)
