# Supplementary material for: Modeling how substitution of sedentary behavior with standing or physical activity is associated with health-related quality of life in colorectal cancer survivors
Source: Cancer Causes Control. 2016 Feb 18;27:513–25. doi: 10.1007/s10552-016-0725-6 (PMC4796364; doi:10.1007/s10552-016-0725-6)
Supplement: Supplementary file 1 — Supplementary material 1 (PDF 147 kb) [file 10552_2016_725_MOESM1_ESM.pdf]

Modeling how substitution of sedentary behavior with standing or physical activity is associated with health-related quality of life in colorectal cancer survivors

Eline H. van Roekel, Martijn J.L. Bours, José J.L. Breedveld-Peters, Paul J.B. Willems, Kenneth Meijer, IJmert Kant, Piet A. van den Brandt, Geerard L. Beets, Silvia Sanduleanu, and Matty P. Weijenberg

Corresponding author: Eline H. van Roekel, Maastricht University, Department of Epidemiology, GROW School for Oncology and Developmental Biology, P.O. Box 616, 6200 MD Maastricht, The Netherlands. Phone: +31 43 38 83428. Fax: +31 43 38 84128. E-mail: eline.vanroekel@maastrichtuniversity.nl.

Cancer Causes & Control

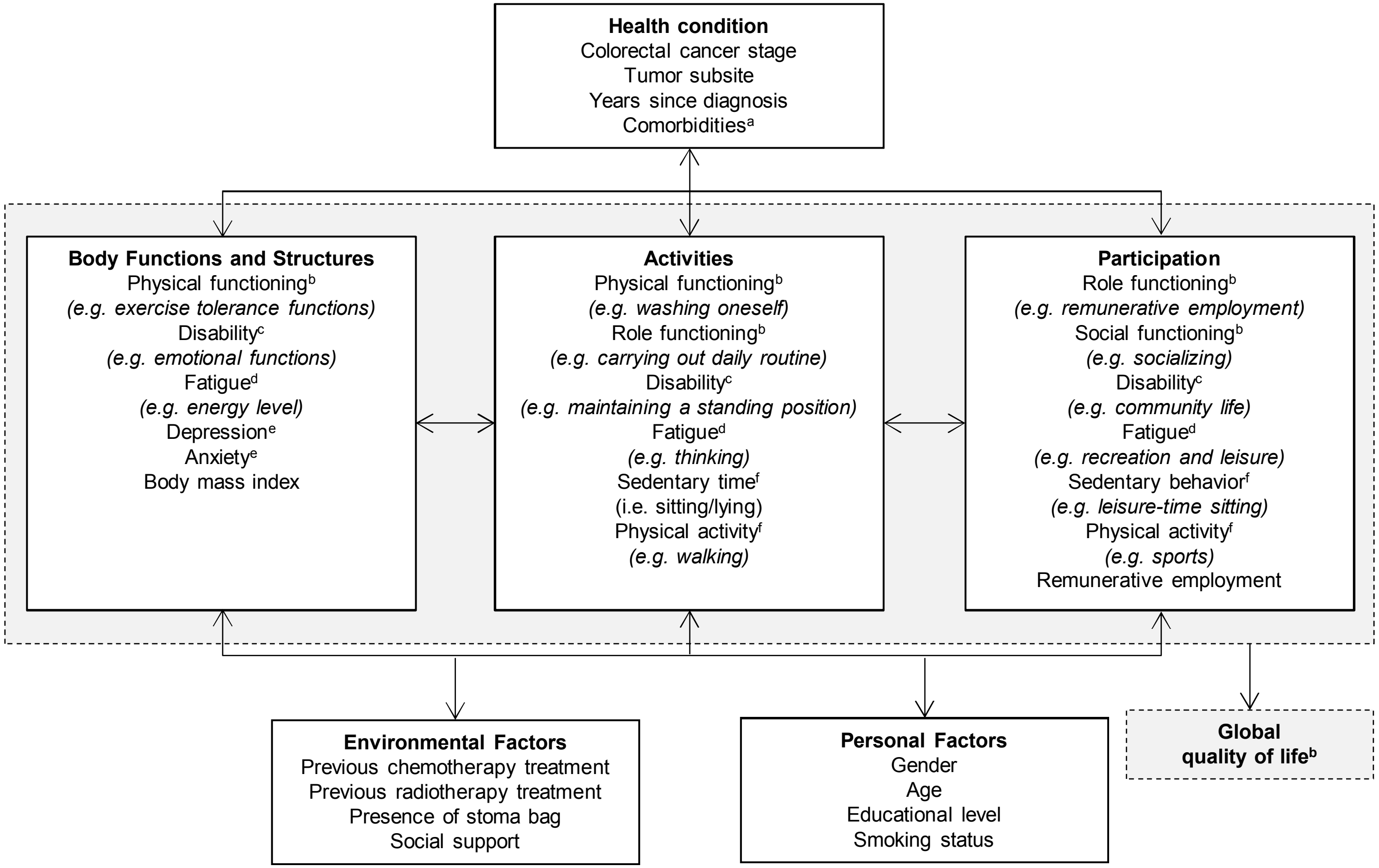

Supplementary Figure 1: Conceptual model, based on the International Classification of Functioning, Disability and Health (ICF), showing relevant factors for studying sedentary behavior and health-related quality of life outcomes in colorectal cancer survivors, which were measured within the cross-sectional component of the Energy for life after ColoRectal cancer study and included in the current analyses.

Footnotes:

The ICF is a biopsychosocial framework of health and functioning that comprehensively describes and classifies functioning of an individual or a group of individuals in a certain health state by differentiating between body functions and structures, activities, and participation, in the context of environmental and personal factors (barriers/facilitators). Certain HRQoL outcomes are shown within multiple ICF domains for functioning (e.g., Physical functioning is shown in both 'Body functions and Structures' and 'Activities'), because meaningful concepts contained in these measures were linked to specific ICF categories within these different domains of functioning (e.g. 'exercise tolerance functions' and 'washing oneself').

Measurement instruments: [a]Self-administered Comorbidity Questionnaire; [b]European Organization for the Research and Treatment of Cancer Quality of Life Questionnaire-Core 30 (EORTC QLQ-C30); [c]12-item World Health Organization Disability Assessment Schedule II; [d]Checklist Individual Strength; [e]Hospital Anxiety and Depression Scale; [f]MOX activity monitor.
