# Supplementary material for: Modeling how substitution of sedentary behavior with standing or physical activity is associated with health-related quality of life in colorectal cancer survivors
Source: Cancer Causes Control. 2016 Feb 18;27:513–25. doi: 10.1007/s10552-016-0725-6 (PMC4796364; doi:10.1007/s10552-016-0725-6)
Supplement: Supplementary file 2 — Supplementary material 2 (PDF 142 kb) [file 10552_2016_725_MOESM2_ESM.pdf]

Modeling how substitution of sedentary behavior with standing or physical activity is associated with health-related quality of life in colorectal cancer survivors

Eline H. van Roekel, Martijn J.L. Bours, José J.L. Breedveld-Peters, Paul J.B. Willems, Kenneth Meijer, IJmert Kant, Piet A. van den Brandt, Geerard L. Beets, Silvia Sanduleanu, and Matty P. Weijenberg

Corresponding author: Eline H. van Roekel, Maastricht University, Department of Epidemiology, GROW School for Oncology and Developmental Biology, P.O. Box 616, 6200 MD Maastricht, The Netherlands. Phone: +31 43 38 83428. Fax: +31 43 38 84128. E-mail: eline.vanroekel@maastrichtuniversity.nl.

Cancer Causes & Control

Supplementary Table 1. Calculated cutoffs for regression coefficients from isotemporal substitution models reported within this article being described as "meaningful", based on minimally important differences in health-related quality of life outcomes[a]

Health-related quality of life outcomes[b]	Cutoff for substituting sedentary time with standing or physical activity	Cutoff for substituting standing time with physical activity
Global quality of life	2.9	3.8
Physical functioning	4.1	5.4
Role functioning	5.6	7.3
Social functioning	3.2	4.2
Disability	2.3	3.0
Fatigue	4.0	5.2
Depression	0.5	0.7
Anxiety	0.5	0.7

[a]Calulation based on definition that regression coefficients from isotemporal substitution models presented in this article were described as "meaningful" if the mean difference in health-related quality of life outcome associated with a difference of 2 standard deviations (SDs) of the substituted activity variable (i.e., sedentary [SD, 1.7 hours/day] or standing [SD, 1.3 hours/day] time) with time in another activity variable (i.e., standing or physical activity) exceeded the minimally important difference cutoff for a medium effect size (according to published guidelines for medium cross-sectional differences in the EORTC QLQ-C30 subscales global health status/quality of life [10], physical functioning [14], role functioning [19], social functioning [11]; or 0.5 times the SD of the outcome score for disability [7.8], fatigue [13.6], depression [1.7], and anxiety [1.8] for which no guidelines are available).

[b]Scales are 0-100 (global quality of life, physical, role and social functioning, and disability), 20-140 (fatigue), and 0-21 (depression and anxiety), with higher scores indicating higher global quality of life, physical, role and social functioning, disability, fatigue, depression and anxiety.

Supplementary Table 2. Analyses of isotemporal substitution models[a] for analyzing associations of substituting sedentary time by standing or physical activity, and for substituting standing time with physical activity, with health-related quality of life scores[b] in colorectal cancer survivors in confounder-adjusted linear regression models[c]

Health-related quality of life outcomes	Substituting 1 hour/day of sedentary time with standing		Substituting 1 hour/day of sedentary time with physical activity		Substituting 1 hour/day of standing time with physical activity	
	β	95% CI	β	95% CI	β	95% CI
Global quality of life (*n* = 136)	1.0	-1.6, 3.5	3.2	-1.6, 7.9	2.2	-3.9, 8.4
Physical functioning (*n* = 136)	3.1	0.5, 5.7	5.6	0.7, 10.6	2.6	-3.9, 9.0
Role functioning (*n* = 136)	3.1	-0.4, 6.6	1.7	-4.9, 8.3	-1.4	-9.9, 7.2
Social functioning (*n* = 136)	1.3	-1.4, 3.9	0.4	-4.6, 5.4	-0.8	-7.3, 5.7
Disability (*n* = 132)	-3.0	-4.9, -1.1	-3.3	-6.9, 0.3	-0.4	-5.0, 4.3
Fatigue (*n* = 134)	-4.0	-7.6, -0.3	-3.6	-10.5, 3.3	0.3	-8.5, 9.2
Depression (*n* = 135)	-0.1	-0.5, 0.4	-0.5	-1.4, 0.4	-0.4	-1.6, 0.7
Anxiety (*n* = 135)	0.0	-0.5, 0.5	-0.7	-1.7, 0.3	-0.7	-2.0, 0.5

Abbreviations: β, unstandardized regression coefficient (representing the difference in mean health-related quality of life score per substitution of 1 hour/day of sedentary or standing time with standing or physical activity, while keeping total time constant); CI, confidence interval.

[a]All activity categories except for the category to be substituted (i.e., sedentary or standing time), and total waking wear time were entered in a single model, to estimate associations of substituting 1 hour/day of sedentary or standing time (the excluded variable) with standing or physical activity, while keeping total time constant.

[b]Scales are 0-100 (global quality of life, physical, role and social functioning, and disability), 20-140 (fatigue), and 0-21 (depression and anxiety), with higher scores indicating higher global quality of life, physical, role and social functioning, disability, fatigue, depression and anxiety.

[c]Models were adjusted for age (years), gender, number of comorbidities (0/1/≥2), smoking status (current/previous or never), time since diagnosis (years), cancer stage (I/II/III), body mass index (kg/m^2), perceived deficiency in social support score (continuous), chemotherapy received (yes/no; only models with physical functioning, fatigue and depression as outcome), stoma (yes/no; only models with physical and role functioning, disability and anxiety as outcome), tumor subsite (colon/rectum, with rectosigmoid classified as rectum; only models with physical and role functioning, and disability as outcome), education level (low/medium/high; only models with fatigue and depression as outcome), having a partner (yes/no; only models with anxiety as outcome).

Supplementary Table 3. Results of logistic isotemporal substitution models with dichotomized health-related quality of life scores[a] in colorectal cancer survivors[b]

Health-related quality of life outcomes	Substituting 1 hour/day of sedentary with standing		Substituting 1 hour/day of sedentary with physical activity		Substituting 1 hour/day of standing with physical activity	
	OR	95% CI	OR	95% CI	OR	95% CI
Global quality of life (*n* = 136)	1.0	0.7, 1.5	1.2	0.6, 2.5	1.2	0.5, 3.1
Physical functioning (*n* = 136)	1.1	0.7, 1.7	1.7	0.8, 3.7	1.5	0.5, 4.3
Role functioning (*n* = 136)	1.2	0.8, 1.8	0.7	0.3, 1.5	0.6	0.2, 1.6
Social functioning (*n* = 136)	1.2	0.8, 1.8	0.6	0.3, 1.3	0.5	0.2, 1.4
Disability (*n* = 132)	0.6	0.4, 0.9	0.9	0.4, 1.9	1.6	0.6, 4.2
Fatigue (*n* = 134)	1.0	0.6, 1.4	0.6	0.3, 1.3	0.7	0.3, 1.7
Depression (*n* = 135)	1.1	0.7, 1.6	0.8	0.4, 1.6	0.8	0.3, 1.9
Anxiety (*n* = 135)	1.1	0.7, 1.5	0.7	0.4, 1.5	0.7	0.3, 1.7

Abbreviations: CI, confidence interval; OR, odds ratio.

[a]Scales are 0-100 (global quality of life, physical, role and social functioning, and disability), 20-140 (fatigue), and 0-21 (depression and anxiety), with higher scores indicating higher global quality of life, physical, role and social functioning, disability, fatigue, depression and anxiety; dichotomized with gender-specific medians as cutoff values for global quality of life (men: ≥83.3 vs <83.3; women: ≥83.3 vs <83.3), physical functioning (men: ≥93.3 vs <93.3; women: ≥80.0 vs <80.0), role functioning (men: 100.0 vs <100.0; women: 100.0 vs <100.0) and social functioning (men: 100.0 vs <100.0; women: 100.0 vs <100.0), disability (men: ≥5.6 vs <5.6; women: ≥11.1 vs <11.1), fatigue (men: ≥54.0 vs <54.0; women: ≥50.0 vs <50.0), depression (men: ≥4.0 vs <4.0; women: ≥4.0 vs <4.0), and anxiety (men: ≥4.0 vs <4.0; women: ≥5.0 vs <5.0); with odds ratios for high vs low levels of quality of life, physical, role and social functioning, and disability, fatigue, depression, and anxiety.

[b]All activity categories except for the category to be substituted (i.e., sedentary or standing time), and total waking wear time were entered in a single model, to estimate associations of substituting 1 hour/day of sedentary or standing time (the excluded variable) with standing or physical activity, while keeping total time constant. Models were adjusted for age (years), gender, number of comorbidities (0/1/≥2), smoking status (current/previous or never), time since diagnosis (years), cancer stage (I/II/III), body mass index (kg/m^2), perceived deficiency in social support score (continuous), chemotherapy received (yes/no; only models with physical functioning, fatigue and depression as outcome), stoma (yes/no; only models with physical and role functioning, disability and anxiety as outcome), tumor subsite (colon/rectum, with rectosigmoid classified as rectum; only models with physical and role functioning, and disability as outcome), education level (low/medium/high; only models with fatigue and depression as outcome), having a partner (yes/no; only models with anxiety as outcome).

Supplementary Table 4. Results of subgroup analyses for associations of substituting sedentary time by standing or physical activity, and for substituting standing time with physical activity, with health-related quality of life scores[a] in colorectal cancer survivors in isotemporal substitution linear regression models[b] stratified for gender, age, number of comorbidities, body mass index and perceived deficiency in social support

	Substituting 1 hour/day of sedentary time with standing		Substituting 1 hour/day of sedentary time with physical activity		Substituting 1 hour/day of standing time with physical activity	
	β	95% CI	β	95% CI	β	95% CI
<u>Gender</u>						
Global quality of life						
Total group (*n* = 136)	1.0	-1.6, 3.5	3.2	-1.6, 7.9	2.2	-3.9, 8.4
Men (*n* = 82)	-0.8	-4.1, 2.4	2.2	-3.8, 8.3	3.1	-5.0, 11.2
Women (*n* = 54)	2.4	-1.8, 6.6	3.7	-4.2, 11.7	1.3	-8.8, 11.5
Physical functioning						
Total group (*n* = 136)	3.1	0.5, 5.7	5.6	0.7, 10.6	2.6	-3.9, 9.0
Men (*n* = 82)	1.3	-2.2, 4.7	0.3	-6.2, 6.9	-0.9	-9.6, 7.7
Women (*n* = 54)	5.2	1.1, 9.3	9.8	2.0, 17.5	4.5	-5.5, 14.5
Role functioning						
Total group (*n* = 136)	3.1	-0.4, 6.6	1.7	-4.9, 8.3	-1.4	-9.9, 7.2
Men (*n* = 82)	-1.2	-5.7, 3.3	2.1	-6.3, 10.5	3.3	-7.9, 14.5
Women (*n* = 54)	5.7	-0.4, 11.7	3.6	-7.7, 15.0	-2.0	-16.7, 12.6
Social functioning						
Total group (*n* = 136)	1.3	-1.4, 3.9	0.4	-4.6, 5.4	-0.8	-7.3, 5.7
Men (*n* = 82)	0.3	-4.4, 5.1	3.1	-5.7, 11.9	2.7	-9.0, 14.5
Women (*n* = 54)	1.3	-1.3, 3.9	-1.5	-6.3, 3.4	-2.8	-9.0, 3.4
Disability						
Total group (*n* = 132)	-3.0	-4.9, -1.1	-3.3	-6.9, 0.3	-0.4	-5.0, 4.3
Men (*n* = 81)	-2.2	-4.7, 0.4	-0.3	-5.0, 4.4	1.8	-4.4, 8.0
Women (*n* = 51)	-3.0	-6.1, 0.1	-7.2	-12.9, -1.4	-4.2	-11.6, 3.3
Fatigue						
Total group (*n* = 134)	-4.0	-7.6, -0.3	-3.6	-10.5, 3.3	0.3	-8.5, 9.2
Men (*n* = 82)	0.2	-4.9, 5.4	-4.2	-13.7, 5.3	-4.4	-16.9, 8.1
Women (*n* = 52)	-5.7	-11.9, 0.6	-3.1	-14.1, 7.8	2.6	-11.5, 16.7
Depression						
Total group (*n* = 135)	-0.1	-0.5, 0.4	-0.5	-1.4, 0.4	-0.4	-1.6, 0.7
Men (*n* = 81)	0.1	-0.6, 0.8	0.4	-1.0, 1.7	0.3	-1.5, 2.0
Women (*n* = 54)	0.2	-0.4, 0.8	-1.0	-2.0, 0.1	-1.2	-2.6, 0.2
Anxiety						
Total group (*n* = 135)	0.0	-0.5, 0.5	-0.7	-1.7, 0.3	-0.7	-2.0, 0.5
Men (*n* = 81)	-0.2	-1.0, 0.6	0.0	-1.5, 1.4	0.1	-1.8, 2.1
Women (*n* = 54)	0.2	-0.5, 0.9	-1.0	-2.4, 0.4	-1.2	-3.0, 0.6
<u>Age</u>						
Global quality of life						
Total group (*n* = 136)	1.0	-1.6, 3.5	3.2	-1.6, 7.9	2.2	-3.9, 8.4
<70 years of age (*n* = 68)	-0.2	-3.8, 3.5	3.3	-3.4, 10.0	3.5	-5.7, 12.6
≥70 years of age (*n* = 68)	2.5	-1.5, 6.6	3.9	-4.4, 12.2	1.3	-8.8, 11.4
Physical functioning						
Total group (*n* = 136)	3.1	0.5, 5.7	5.6	0.7, 10.6	2.6	-3.9, 9.0
<70 years of age (*n* = 68)	6.2	2.3, 10.0	1.5	-5.4, 8.4	-4.6	-14.1, 4.9
≥70 years of age (*n* = 68)	1.4	-2.6, 5.3	6.8	-1.6, 15.3	5.5	-4.8, 15.7
Role functioning						
Total group (*n* = 136)	3.1	-0.4, 6.6	1.7	-4.9, 8.3	-1.4	-9.9, 7.2
<70 years of age (*n* = 68)	3.9	-1.3, 9.2	1.8	-7.6, 11.2	-2.1	-15.1, 10.8
≥70 years of age (*n* = 68)	2.7	-2.8, 8.1	-0.2	-11.7, 11.3	-2.9	-17.0, 11.2
Social functioning						
Total group (*n* = 136)	1.3	-1.4, 3.9	0.4	-4.6, 5.4	-0.8	-7.3, 5.7
<70 years of age (*n* = 68)	1.0	-2.6, 4.5	-1.0	-7.5, 5.5	-2.0	-10.8, 6.9
≥70 years of age (*n* = 68)	0.4	-4.0, 4.7	2.6	-6.2, 11.4	2.2	-8.5, 12.9

(Table continues on next page)

	Substituting 1 hour/day of sedentary time with standing		Substituting 1 hour/day of sedentary time with physical activity		Substituting 1 hour/day of standing time with physical activity	
	β	95% CI	β	95% CI	β	95% CI
Disability						
Total group (*n* = 132)	-3.0	-4.9, -1.1	-3.3	-6.9, 0.3	-0.4	-5.0, 4.3
<70 years of age (*n* = 68)	-4.2	-7.0, -1.4	-2.1	-7.1, 2.9	2.1	-4.9, 9.0
≥70 years of age (*n* = 64)	-2.0	-5.0, 1.0	-2.5	-8.8, 3.9	-0.5	-8.3, 7.4
Fatigue						
Total group (*n* = 134)	-4.0	-7.6, -0.3	-3.6	-10.5, 3.3	0.3	-8.5, 9.2
<70 years of age (*n* = 66)	-3.1	-9.2, 3.0	-5.5	-16.3, 5.3	-2.4	-17.2, 12.3
≥70 years of age (*n* = 68)	-5.3	-10.6, -0.0	-1.5	-12.4, 9.3	3.8	-9.3, 16.9
Depression						
Total group (*n* = 135)	-0.1	-0.5, 0.4	-0.5	-1.4, 0.4	-0.4	-1.6, 0.7
<70 years of age (*n* = 68)	-0.1	-0.8, 0.6	-0.7	-2.0, 0.6	-0.6	-2.4, 1.2
≥70 years of age (*n* = 67)	-0.1	-0.8, 0.7	-0.3	-1.9, 1.3	-0.2	-2.1, 1.7
Anxiety						
Total group (*n* = 135)	0.0	-0.5, 0.5	-0.7	-1.7, 0.3	-0.7	-2.0, 0.5
<70 years of age (*n* = 68)	-0.1	-0.8, 0.6	-0.6	-1.9, 0.7	-0.5	-2.3, 1.3
≥70 years of age (*n* = 67)	0.0	-0.8, 0.8	-0.9	-2.6, 0.9	-0.9	-3.0, 1.2
<u>Number of comorbidities</u>						
Global quality of life						
Total group (*n* = 136)	1.0	-1.6, 3.5	3.2	-1.6, 7.9	2.2	-3.9, 8.4
<2 comorbidities (*n* = 64)	0.1	-3.0, 3.2	4.3	-0.9, 9.5	4.2	-2.9, 11.2
≥2 comorbidities (*n* = 72)	1.6	-2.6, 5.7	2.0	-7.1, 11.0	0.4	-10.8, 11.6
Physical functioning						
Total group (*n* = 136)	3.1	0.5, 5.7	5.6	0.7, 10.6	2.6	-3.9, 9.0
<2 comorbidities (*n* = 64)	3.0	-0.9, 6.9	4.1	-2.4, 10.5	1.1	-7.7, 9.9
≥2 comorbidities (*n* = 72)	2.5	-1.2, 6.2	5.1	-3.9, 14.1	2.6	-8.1, 13.3
Role functioning						
Total group (*n* = 136)	3.1	-0.4, 6.6	1.7	-4.9, 8.3	-1.4	-9.9, 7.2
<2 comorbidities (*n* = 64)	3.4	-1.0, 7.8	4.1	-3.2, 11.3	0.7	-9.2, 10.6
≥2 comorbidities (*n* = 72)	2.9	-2.5, 8.4	-7.9	-20.7, 4.8	-10.9	-26.1, 4.4
Social functioning						
Total group (*n* = 136)	1.3	-1.4, 3.9	0.4	-4.6, 5.4	-0.8	-7.3, 5.7
<2 comorbidities (*n* = 64)	1.4	-1.2, 3.9	-1.0	-5.3, 3.2	-2.4	-8.2, 3.3
≥2 comorbidities (*n* = 72)	2.0	-2.5, 6.5	0.8	-8.9, 10.4	-1.2	-13.3, 10.8
Disability						
Total group (*n* = 132)	-3.0	-4.9, -1.1	-3.3	-6.9, 0.3	-0.4	-5.0, 4.3
<2 comorbidities (*n* = 64)	-2.9	-5.3, -0.5	-2.4	-6.4, 1.5	0.5	-4.9, 5.9
≥2 comorbidities (*n* = 68)	-3.3	-6.4, -0.3	-2.2	-9.2, 4.8	1.2	-7.3, 9.7
Fatigue						
Total group (*n* = 134)	-4.0	-7.6, -0.3	-3.6	-10.5, 3.3	0.3	-8.5, 9.2
<2 comorbidities (*n* = 64)	-1.7	-7.2, 3.8	-3.8	-13.2, 5.5	-2.1	-14.7, 10.5
≥2 comorbidities (*n* = 70)	-5.3	-11.1, 0.4	-3.7	-15.7, 8.3	1.7	-13.2, 16.5
Depression						
Total group (*n* = 135)	-0.1	-0.5, 0.4	-0.5	-1.4, 0.4	-0.4	-1.6, 0.7
<2 comorbidities (*n* = 63)	0.1	-0.6, 0.8	-0.3	-1.5, 0.9	-0.4	-2.1, 1.2
≥2 comorbidities (*n* = 72)	-0.1	-0.8, 0.7	-1.1	-2.6, 0.5	-1.0	-2.9, 0.9
Anxiety						
Total group (*n* = 135)	0.0	-0.5, 0.5	-0.7	-1.7, 0.3	-0.7	-2.0, 0.5
<2 comorbidities (*n* = 64)	0.6	-0.1, 1.4	-0.9	-2.1, 0.4	-1.5	-3.2, 0.2
≥2 comorbidities (*n* = 71)	-0.5	-1.1, 0.2	0.3	-1.2, 1.8	0.8	-1.0, 2.6
<u>Body mass index</u>						
Global quality of life						
Total group (*n* = 136)	1.0	-1.6, 3.5	3.2	-1.6, 7.9	2.2	-3.9, 8.4
BMI<30 kg/m^2 (*n* = 98)	0.2	-2.4, 2.8	6.5	1.2, 11.8	6.3	-0.6, 13.1
BMI≥30 kg/m^2 (*n* = 38)	-0.2	-7.2, 6.9	1.8	-9.4, 13.0	2.0	-12.4, 16.4

(Table continues on next page)

	Substituting 1 hour/day of sedentary time with standing		Substituting 1 hour/day of sedentary time with physical activity		Substituting 1 hour/day of standing time with physical activity	
	β	95% CI	β	95% CI	β	95% CI
Physical functioning						
Total group (*n* = 136)	3.1	0.5, 5.7	5.6	0.7, 10.6	2.6	-3.9, 9.0
BMI<30 kg/m^2 (*n* = 98)	2.7	-0.3, 5.7	6.0	-0.2, 12.2	3.3	-4.7, 11.3
BMI≥30 kg/m^2 (*n* = 38)	4.0	-3.9, 11.8	7.4	-7.0, 21.7	3.4	-14.0, 20.8
Role functioning						
Total group (*n* = 136)	3.1	-0.4, 6.6	1.7	-4.9, 8.3	-1.4	-9.9, 7.2
BMI<30 kg/m^2 (*n* = 98)	2.3	-1.5, 6.2	7.8	-0.0, 15.7	5.5	-4.7, 15.7
BMI≥30 kg/m^2 (*n* = 38)	1.6	-9.1, 12.2	-9.3	-24.2, 5.5	-10.9	-30.6, 8.8
Social functioning						
Total group (*n* = 136)	1.3	-1.4, 3.9	0.4	-4.6, 5.4	-0.8	-7.3, 5.7
BMI<30 kg/m^2 (*n* = 98)	0.8	-2.4, 4.1	2.3	-4.2, 8.8	1.5	-6.9, 9.9
BMI≥30 kg/m^2 (*n* = 38)	2.9	-3.8, 9.5	-2.3	-12.9, 8.4	-5.1	-18.8, 8.6
Disability						
Total group (*n* = 132)	-3.0	-4.9, -1.1	-3.3	-6.9, 0.3	-0.4	-5.0, 4.3
BMI<30 kg/m^2 (*n* = 96)	-2.9	-5.1, -0.7	-4.3	-8.8, 0.2	-1.4	-7.3, 4.5
BMI≥30 kg/m^2 (*n* = 36)	-1.9	-7.9, 4.1	-3.7	-11.9, 4.4	-1.8	-12.8, 9.2
Fatigue						
Total group (*n* = 134)	-4.0	-7.6, -0.3	-3.6	-10.5, 3.3	0.3	-8.5, 9.2
BMI<30 kg/m^2 (*n* = 96)	-3.2	-7.2, 0.8	-7.9	-15.8, 0.1	-4.7	-15.0, 5.6
BMI≥30 kg/m^2 (*n* = 38)	-6.2	-18.3, 5.8	-0.5	-21.0, 20.0	5.7	-19.0, 30.5
Depression						
Total group (*n* = 135)	-0.1	-0.5, 0.4	-0.5	-1.4, 0.4	-0.4	-1.6, 0.7
BMI<30 kg/m^2 (*n* = 97)	0.2	-0.3, 0.7	-1.5	-2.4, -0.5	-1.7	-2.9, -0.5
BMI≥30 kg/m^2 (*n* = 38)	-0.4	-2.1, 1.3	0.0	-2.8, 2.9	0.4	-3.0, 3.8
Anxiety						
Total group (*n* = 135)	0.0	-0.5, 0.5	-0.7	-1.7, 0.3	-0.7	-2.0, 0.5
BMI<30 kg/m^2 (*n* = 98)	0.3	-0.3, 0.9	-1.5	-2.8, -0.3	-1.8	-3.4, -0.3
BMI≥30 kg/m^2 (*n* = 37)	-0.5	-1.6, 0.5	0.7	-0.9, 2.3	1.3	-0.9, 3.5
Perceived deficiency in social support[c]						
Global quality of life						
Total group (*n* = 136)	1.0	-1.6, 3.5	3.2	-1.6, 7.9	2.2	-3.9, 8.4
No deficiency (*n* = 76)	-3.1	-7.1, 0.9	2.3	-4.5, 9.1	5.4	-3.7, 14.4
Deficiency (*n* = 60)	4.1	0.5, 7.8	3.7	-4.5, 12.0	-0.4	-10.6, 9.8
Physical functioning						
Total group (*n* = 136)	3.1	0.5, 5.7	5.6	0.7, 10.6	2.6	-3.9, 9.0
No deficiency (*n* = 76)	2.0	-1.4, 5.5	4.5	-1.3, 10.2	2.4	-5.2, 10.0
Deficiency (*n* = 60)	4.3	0.2, 8.5	5.8	-3.5, 15.0	1.4	-10.0, 12.9
Role functioning						
Total group (*n* = 136)	3.1	-0.4, 6.6	1.7	-4.9, 8.3	-1.4	-9.9, 7.2
No deficiency (*n* = 76)	-0.9	-5.5, 3.8	-2.4	-10.2, 5.4	-1.6	-11.9, 8.8
Deficiency (*n* = 60)	4.6	-0.8, 10.0	9.6	-2.5, 21.7	5.0	-10.0, 20.0
Social functioning						
Total group (*n* = 136)	1.3	-1.4, 3.9	0.4	-4.6, 5.4	-0.8	-7.3, 5.7
No deficiency (*n* = 76)	-0.0	-3.7, 3.6	0.6	-5.5, 6.8	0.7	-7.5, 8.8
Deficiency (*n* = 60)	2.0	-2.7, 6.6	0.0	-10.6, 10.6	-1.9	-15.0, 11.1
Disability						
Total group (*n* = 132)	-3.0	-4.9, -1.1	-3.3	-6.9, 0.3	-0.4	-5.0, 4.3
No deficiency (*n* = 76)	-0.7	-3.1, 1.8	-3.1	-7.2, 1.0	-2.4	-7.9, 3.0
Deficiency (*n* = 56)	-4.2	-7.1, -1.3	-5.0	-11.6, 1.7	-0.8	-9.0, 7.5
Fatigue						
Total group (*n* = 134)	-4.0	-7.6, -0.3	-3.6	-10.5, 3.3	0.3	-8.5, 9.2
No deficiency (*n* = 76)	-1.0	-6.8, 4.7	-3.5	-13.3, 6.3	-2.5	-15.4, 10.5
Deficiency (*n* = 58)	-8.2	-13.4, -3.0	-1.2	-11.9, 9.5	7.0	-6.4, 20.4

(Table continues on next page)

	Substituting 1 hour/day of sedentary time with standing		Substituting 1 hour/day of sedentary time with physical activity		Substituting 1 hour/day of standing time with physical activity	
	β	95% CI	β	95% CI	β	95% CI
Depression						
Total group (*n* = 135)	-0.1	-0.5, 0.4	-0.5	-1.4, 0.4	-0.4	-1.6, 0.7
No deficiency (*n* = 75)	0.3	-0.3, 1.0	-0.6	-1.7, 0.6	-0.9	-2.3, 0.6
Deficiency (*n* = 60)	-0.4	-1.2, 0.4	0.1	-1.6, 1.8	0.5	-1.6, 2.6
Anxiety						
Total group (*n* = 135)	0.0	-0.5, 0.5	-0.7	-1.7, 0.3	-0.7	-2.0, 0.5
No deficiency (*n* = 76)	0.5	-0.2, 1.2	-1.1	-2.2, 0.1	-1.5	-3.1, -0.0
Deficiency (*n* = 59)	-0.3	-1.1, 0.5	-0.4	-2.4, 1.5	-0.1	-2.5, 2.3

Abbreviations: β, unstandardized regression coefficient (representing the difference in mean health-related quality of life score per substitution of 1 hour/day of sedentary or standing time with standing or physical activity, while keeping total time constant); CI, confidence interval.

[a]Scales are 0-100 (global quality of life, physical, role and social functioning, and disability), 20-140 (fatigue), and 0-21 (depression and anxiety), with higher scores indicating higher global quality of life, physical, role and social functioning, disability, fatigue, depression and anxiety.

[b]All activity categories except for the category to be substituted (i.e., sedentary or standing time), and total waking wear time were entered in a single model, to estimate associations of substituting 1 hour/day of sedentary or standing time (the excluded variable) with standing or physical activity, while keeping total time constant. Models were adjusted for age (years), gender, number of comorbidities (0/1/≥2) , smoking status (current/previous or never), time since diagnosis (years), cancer stage (I/II/III), body mass index (kg/m^2), perceived deficiency in social support score (continuous), chemotherapy received (yes/no; only models with physical functioning, fatigue and depression as outcome), stoma (yes/no; only models with physical and role functioning, disability and anxiety as outcome), tumor subsite (colon/rectum, with rectosigmoid classified as rectum; only models with physical and role functioning, and disability as outcome), education level (low/medium/high; only models with fatigue and depression as outcome), having a partner (yes/no; only models with anxiety as outcome).

[c]Dichotomized based on 6-item Social Support List score (scale: 6-18, with higher score indicating higher deficiency); categorized into no deficiency (score=6) vs deficiency (score>6).
